# Assessment and model guided cancer screening promotion by village doctors in China: a randomized controlled trial protocol

**DOI:** 10.1186/s12885-015-1688-9

**Published:** 2015-10-12

**Authors:** Rui Feng, Xingrong Shen, Jing Chai, Penglai Chen, Jing Cheng, Han Liang, Ting Zhao, Rui Sha, Kaichun Li, Debin Wang

**Affiliations:** 1 Department of Literature Review and Analysis, Library of Anhui Medical University, Hefei, Anhui China; 2 School of Health Service Management, Anhui Medical University, Hefei, Anhui China; 3 Luan Center for Disease Control and Prevention, Luan, Anhui China; 4 Collaboration Center for Cancer Control, First Affiliated Hospital of Anhui Medical University, Hefei, Anhui China

**Keywords:** Cancer, Screening uptake, Randomized controlled trial, Prevention and treatment integration

## Abstract

**Background:**

Proven cost-effectiveness contrasted by low uptake of cancer screening (CS) calls for new methodologies promoting the service. Contemporary interventions in this regard relies primarily on strategies targeting general or specific groups with limited attention being paid to individualized approaches. This trial tests a novel package promoting CS utilization via continuous and tailored counseling delivered by primary caregivers. It aims at demonstrating that high risk individuals in the intervention arm will, compared to those in the delayed intervention condition, show increased use of CS service.

**Methods/Design:**

The trial adopts a quasi-randomized controlled trial design and involves 2160 high risk individuals selected, via rapid and detailed risk assessments, from about 72,000 farmers aged 35+ in 36 administrative villages randomized into equal intervention and delayed intervention arms. The CS intervention package uses: a) village doctors and village clinics to deliver personalized and thus relatively sophisticated CS counseling; b) two-stage risk assessment models in identifying high risk individuals to focus the intervention on the most needed; c) standardized operation procedures to guide conduct of counseling; d) real-time effectiveness and quality monitoring to leverage continuous improvement; e) web-based electronic system to enable prioritizing complex determinants of CS uptake and tailoring counseling sessions to the changing needs of individual farmers. The intervention arm receives baseline and semiannual follow up evaluations plus CS counseling for 5 years; while the delayed intervention arm, only the same baseline and follow-up evaluations for the first 5 years and CS counseling starting from the 6th year if the intervention proved effective. Evaluation measures include: CS uptake by high risk farmers and changes in their knowledge, perceptions and self-efficacy about CS.

**Discussion:**

Given the complexity and heterogeneity in the determinant system of individual CS service seeking behavior, personalized interventions may prove to be an effective strategy. The current trial distinguishes itself from previous ones in that it not only adopts a personalized strategy but also introduces a package of pragmatic solutions based on proven theories for tackling potential barriers and incorporating key success factors in a synergetic way toward low cost, effective and sustainable CS promotion.

**Trial registration:**

ISRCTN33269053

**Electronic supplementary material:**

The online version of this article (doi:10.1186/s12885-015-1688-9) contains supplementary material, which is available to authorized users.

## Background

Cancer has become one of the most serious chronic diseases worldwide [1]. Steadily growing new cases, high mortality rate combined with lack of radical cures have made prevention and early diagnosis priority strategies for stemming the epidemic [2–5]. Numerous studies suggest that cancer screening (CS) is cost-effective in shortening delay for treatment, prolonging survival time and improving quality of life [6–8]. However, uptake of CS is rather low [9, 10]. This is especially true in China. Wang et al. examined screening uptake by 53,513 women using 2010 China Chronic Disease and Risk Factor Surveillance data and found that only 21.9 % of them reported use of breast CS [11]. Similarly, a survey of 711,243 women aged from 25 through to 65 in the pilot areas of a cervical CS project in Beijing revealed that only 20.94 % had used the service [12]. Low uptake of screening services is even more prevalent in resource-poor rural China where over 75 % of the nation’s vast population lives [13]. Meng et al. reported that utilization rates of cervical and breast CS was 9.0 and 6.2 % respectively in rural China compared with 25.1 and 28.1 % of that in urban areas [14].

Low CS uptake has been attributed to a whole range of factors. Many studies have shown that use of CS is linked with age, gender, family history, culture, knowledge, education, location, occupation, language barriers and others [15–18]. Fears about over-diagnosis of disease, inaccurate test results, burden of disease labeling and side effects of treatment also affect decision on seeking CS [19, 20]. Perhaps the biggest obstacle to uptake relates to the complexity of factors and their interactions involved in the paths from risks to cancer onset and harms and from CS pre-ideation to uptake [21]. This complexity makes it hard for ordinary residents to perceive cause-effect relationships between risks versa cancers and CS versa harm reduction and thus greatly weakens their desire to seek CS [22]. It also explains, to a large extent, why the effect of general or non-tailored interventions (like public education programs) often falls far from expected [22, 23]. Because promoting desired CS uptake relies heavily on leveraging multiple factors within the complicated determinant system of the behavior in a synergetic way; and this is to the disadvantage of general “education” and often beyond the ability of ordinary people especially old rural farmers with high illiteracy [22]. Personalized promotion may prove to be an effective solution since it allows for identifying limited critical influence factors and paths from a large amount of potential alternatives and thus forming tailored approaches for the specific individual under concern, rather than general education for whole or a segment of promotion [24]. Primary care settings provide an ideal place for implementing such personalized screening population. However, most primary care givers are not fully prepared for delivering CS. This applies especially to resource-poor rural China [25].

Based on the above considerations, this study tests an novel personalized intervention package for promoting CS utilization. In essence, the package tries to tackle main barriers and incorporate key success factors to desired CS uptake in a synergetic way toward cost-effectiveness and long-term sustainability. It: a) choses village doctors as a key solution to the widespread lack of professional manpower in implementing personalized, continuous and thus relatively sophisticated screening promotion; b) uses two-stage risk assessment models in identifying high risk individuals so as to greatly narrow down the scale of intervention and focus scarce resources on the most needed; c) applies standardized operation procedures (SOPs) derived from proven theories and best practices in simplifying and smoothing screening promotion yet ensuring delivery of essential steps and key success elements; d) employs a real-time effectiveness and quality monitoring in leveraging continuous CS counseling improvement; e) utilizes powerful recording, retrieving and processing abilities of computer systems to enable prioritizing complex determinants of screening uptake, linking counseling sessions happened at different time points and hence delivering highly coordinated intervention.

This study is designed and implemented as an integral part of an umbrella project which uses a intervention package called eCROPS-CA [22]. Here, CA stands for cancer and eCROPS, for electronic supports and supervision (e), counseling cancer prevention (C), recipe for objective behaviors (R), operational toolkit (O), performance-based incentives (P), and screening and assessment (S) respectively. The primary objective of this umbrella project is reducing the incidence rate of leading cancers among high risk farmers in rural China by means of promoting a set of pre-determined objective behaviors including improving diet and nutrition, increasing physical activity, reducing risk behaviors, avoiding environmental carcinogens, treating cancer-related conditions, seeking regular CS, and involving relatives and friends. This paper focuses on regular CS uptake, one of the objective behaviors of eCROPS-CA. It not only sheds new lights on promoting CS via routine primary care but also provides as an example showing how individual objective behaviors within eCROPS-CA are realized.

## Aims/Objectives

The study aims at demonstrating that the aforementioned intervention package is effective in leveraging CS uptake and high risk individuals in the intervention arm will, compared to those in the delayed intervention condition, show increased use of screening service and improved KAP (knowledge, attitudes and practices) in relation to CS.

## Methods

### Study design

The study adopts a quasi-randomized controlled trial (RCT) design involving some 2160 high risk individuals randomized into equal intervention and delayed intervention arms. The intervention arm receives baseline and semiannual follow up evaluations plus personalized CS counseling and different combinations of counseling sessions for other objective behaviors for 5 years; while the delayed intervention arm, only the same baseline and follow up evaluations for the first 5 years and the same CS counseling starting from year 6 if the intervention is proved effective.

#### Eligibility criteria

Being a sub-trial, the study utilizes a subsample of its umbrella project participants. So the eligibility criteria for recruiting participants in the umbrella project all apply to this trial. These are male and female farmers who: a) are 35 years or older; b) live in the selected villages for over 6 months per year; c) meet the cut point score of RRA (≥ the value of the 70th percentile RRA score) and DRA (≥ the value of the 80th percentile DRA score); d) have not yet diagnosed with cancer(s) or mental illness or other serious illness or disability that prevent them from attending planed counseling sessions. In addition, participants in this sub-trial should also meet the standards for CS set by China National Center for Diseases Prevention and Control (CDC) [26].

#### Selection of participants

This sub-trial does not incur recruitment of additional participants, since the sample size needed for checking the expected key assumption of this trial, CS uptake is higher in the intervention arm than in the delayed-intervention arm, is smaller than that of its umbrella trial, eCROPS-CA prevents leading cancers and results in incidence differences between the two arms. As described in our previous paper, eCROPS-CA recruits 4320 high risk individuals selected, via RRA and DRA, from about 72,000 farmers aged 35 or older in 36 administrative villages determined through a clustered randomization process [22]. Given this, all those who are enrollees of eCROPS-CA and also meet the CS standards set by China CDC are treated as the participants of this sub-trial. Therefore, sample size of this sub-trial is estimated as 2160 consisting of 1080 in the intervention and delayed intervention arms respectively (for more information about sampling, please refer to Additional file 1).

### Intervention

#### Framework and profile of CS determinants

**Fig. 1:**
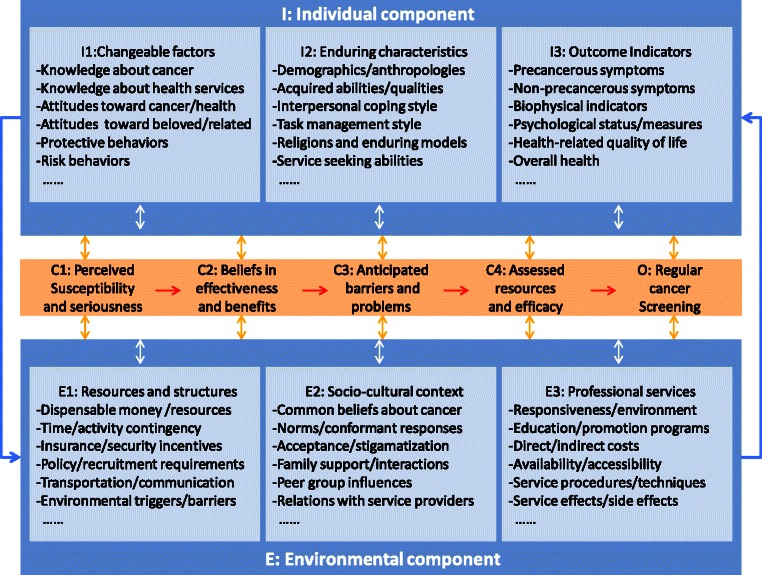
Trans-theoretical framework of cancer screening behavior

The CS promotion package is based on a trans-theoretical framework derived from: a) proven behavior theories including cognitive dissonance, self-efficacy and empathic processes [27]; b) soft systems thinking; and c) consensus group consensus (Fig. 1). Located at the center of the framework is the ultimate goal of this study, optimal CS uptake (O), and its immediate cognitive-affective drivers including perceived susceptibility and seriousness of cancer (C1), beliefs in effectiveness and benefits of CS (C2), anticipated barriers and problems practicing CS (C3) and assessed resources and self-efficacy for overcoming the barriers/problems (C4). These cognitive-affective determinants incorporate several popular behavior theories including health belief model [28], self-efficacy [29], and cognitive dissonance [30]. The paths from C1 through C4 toward CS are influenced by a whole range of individual (I) and environmental (E) factors. And I consists of I1 (relatively easy to change factors), I2 (enduring or hard to change characters) and I3 (outcome variables); while E comprises E1 (resources and structures), E2 (socio-cultural context) and E3 (professional health services). Listed under each of the I/E subareas are six most important determinants of C and ultimately O, e.g., knowledge about cancer, attitudes toward beloved, and protective behaviors under domain I1 and common beliefs about cancer, norms and conformant responses under domain E2.

**Fig. 2:**
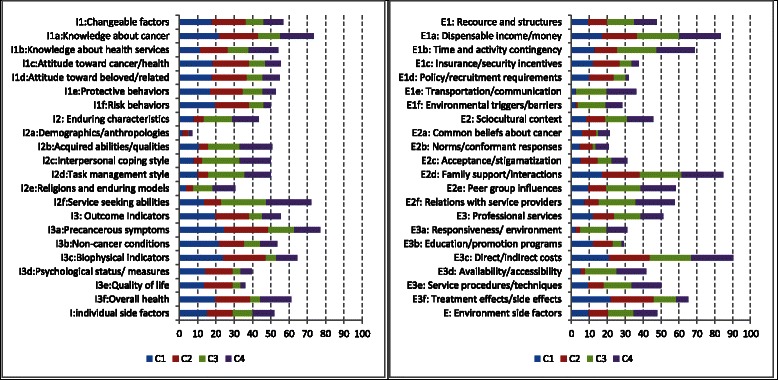
Determinant profile of screening behavior derived via in depth interviews with local farmers (C1, C2, C3 and C4 stand for perceived susceptibility and seriousness, beliefs in effectiveness and benefits, anticipated barriers and problems, and assessed resources and efficacy respectively)

Figure 2 depicts a profile, in terms of the ratings of relative importance, of the determining factors of CS uptake based on the above framework and our qualitative interviews with high risk farmers (*N* = 53) from the planned study sites using the same methods described elsewhere [21]. As the figure shows, putting together, all the individual domain factors (I) gained an average score of 51.9; while the environmental domain factors (E), 48.1. These indicate that individual side factors exert relatively greater effects on CS uptake by the farmers than environment side factors. Similarly, specific factors that plays the most important role in determining CS service seeking is direct and indirect costs of cancer (E3c = 90.3), followed by family support and interactions (E2d = 84.7), dispensable income and money (E1a = 83.3), precancerous symptoms (I3a = 77.1), knowledge about cancer (I1a = 73.6) and health service seeking abilities (I2f = 72.2).

#### Standard operation procedures

All CS counseling sessions utilizes standard operation procedures (SOPs) to ensure delivery of key elements, though the counselor village doctors are encouraged to make the best use of their own experiences. Development of the SOPs employs similar steps and methods we used in deriving the SOPs for diabetes prevention [31, 32]. The aforementioned framework and profile play an important role in the SOP development. Both the guiding principles (Table 1) and detailed content (Table 2) of CS counseling derive from the proven behavior theories and influencing factors incorporated in the framework. For example, steps 1 through 4 of the SOPs for initial counseling (Table 2) are designed to enhance the immediate cognitive-affective derivers (C1 through to C4) in the framework (Figs. 1 and 2) respectively. Similarly, specific items listed under a given step (say step 1) forming the SOPs in Table 2 are designed to address the top ten most influential factors, according to the profile (Fig. 2), of the corresponding immediate cognitive-affective driver (say C1). These arrangements should ensure that the counseling focuses on most important aspects of CS uptake.

**Table 1:** Principles guiding conduct of cancer screening counseling derived from proven theories

Critical points of guiding theories	Principles for counseling cancer screening (CS)
Cognitive dissonance	
-Cognitive dissonance is the feeling of psychological discomfort produced by the combined presence of two thoughts that do not follow from one another;	-Produce a dissonant state about cancer and then controls the direction chosen for the dissonance resolution through skilled use of counseling techniques;
-Being psychologically uncomfortable, the existence of dissonance motivates the person to reduce the dissonance and leads to avoidance of information likely to increase the dissonance;	-View ambivalence as not a barrier but a crucial entry point and can be resolved;
-The greater the discomfort is, the greater the desire to reduce the dissonance of the two cognitive elements;	-Elicit the patient’s desires, expectations, beliefs, fears, and hopes, with particular emphasis on the inconsistencies between these and CS;
-Cognitive dissonance about health derives from perceived susceptibility and seriousness of health problems, benefits and effectiveness of behavior change, barriers and efficacy for implementing the change.	-Address all (rather than part) of critical determinants of CS uptake and discuss risk and harms of cancer, effectiveness and benefits of CS, potential barriers and problems to CS, and strategies, tips and resources for overcoming these barriers and problems.
Self-efficacy	
-Self-efficacy is a person’s belief that he/she can carry out and succeed at a specific change strategy;	-Respect the patient’s autonomy and rely on his/her own capacities to seek CS.
-People with high efficacy expect to succeed, realize favorable outcomes and vice versa;	-Affirm the patient’s freedom of choice and self-direction.
-People with high efficacy believe that they can overcome obstacles by persevering and by improving self-management skills and they do not give up, but rather “stay the course” in the face of difficulties;	-Ensure that motivation to change is elicited from the patient, rather than imposed from outside;
	-Monitor the patient’s motivation and readiness for CS uptake and avoid harsh action plans;
-People with low efficacy believe that their efforts in the face of difficulties will fail and would therefore be a waste of time to undertake and they quickly give up trying.	-Help the patient to verbalize arguments for CS and develop, when ready, a specific plan to utilize CS;
	-Offer advice/supports tailored to anticipated barriers or needs for the patient to seek CS.
Accurate sympathy	
-Accurate empathy defines skillful reflective listening that clarifies and amplifies the participant’s own experience and meaning, without imposing the counselor’s own material;	-Communicate respect and caring, and builds a working alliance between counselor and participant;
	-Encourage the patient to keep talking and exploring key topics, especially ambivalence, about CS,;
-It builds mutual trust between the counselor and participant, enables eliciting true reasons for ambivalence, and enhances participant’s compliance with planned CS uptake.	-Clarify exactly what the patient means and express acceptance and affirmation;
	-Seek to understand the patient’s frame of reference, particularly through reflective listening.

**Table 2:** Checklist of topics to be discussed during initial counseling for cancer screening

Step 1: Counseling awareness of susceptibility and seriousness (C1)		
S1a	Have you ever heard of cancer and how harmful is it?	
□	□	It damages the organ it originates first.
□	□	It then metastases and invades various organs like the lung, brain, liver, bone etc.
□	□	It can cause various physical sufferings like pain, dysfunction, wasting syndrome etc.
□	□	It can cause various psychological sufferings like fears, anxiety, depression etc.
□	□	There are no-radical cures for most cancers and the disease has a high mortality.
□	□	Most cancer therapies are costly and have side effects.
□	□	It affects one’s work, study and business pursues.
□	□	It incurs economic burdens and psychological sufferings on family members and the beloved.
□	□	It may damage family relations.
□	□	It damages one’s image among and expectations by others.
□	□	Other (please enter)
S1b	How, do you think, are the chances for a general farmer in China to get cancer?	
□	□	It’s easy to name friends/acquaintances diagnosed with cancer
□	□	Everyone is susceptible to cancer
□	□	Each year, 300 out of 100 thousand farmers get cancer
□	□	One’s life time chances for getting cancer estimates over 21 %
□	□	Other (please enter)
S1c	How do you think of your own chances to get cancer?	
□	□	I/You have elevated chances for getting [gastric] cancer.
□	□	My/Your latest cancer risk score is [92]
□	□	It ranks top 6 % among all farmers age 35 years and older.
□	□	[I/You have an elder brother who had diagnosed with gastric cancer].
□	□	[I/You have been suffering from chronic gastritis for 27 years].
□	□	[I/You have been eating cured meat and vegetables most frequently for 55 years].
□	□	[I/You have been a heavy alcohol drinker for 40 years].
□	□	[I/You have been smoking about 30 cigarettes a day for 45 years].
□	□	[I/You have been suffering from chronic gastritis for over 20 years].
□	□	[I/You have been feeling decreasing appetite for the last 3 years].
□	□	Other (please enter)
Step 2: Counseling beliefs in effectiveness and benefits of CS (C2)		
S2a	What, do you think, you can get from cancer screening?	
□	□	Most cancers develop through a long-period of pre-cancerous conditions [like polyps, atrophic gastritis].
□	□	These pre-cancerous conditions can be corrected at a minimum cost.
□	□	Cancer screening can detect and correct the pre-cancerous conditions and thus prevent cancers.
□	□	After onset, cancer proliferates and damages human body at an escalating speed.
□	□	At early stages, cancer cells confine within limited boundary and can be radically cleared, e.g., by surgery.
□	□	At late stages, cancer cells metastases to other organs and becomes hard to be cleared from human body.
□	□	When specific symptoms are felt, cancer has generally developed into quite late a stage.
□	□	The earlier the detection of cancer, the better the outcomes of cancer treatment.
□	□	Regular screening not only detects early cancer but also communicates knowledge about cancer.
□	□	Cancer screening also helps in finding and correcting other health problems.
□	□	Other (please enter)
Step 3: Counseling anticipation of barriers and problems (C3)		
S3a	What problems or barriers you may encounter in seeking cancer screening?	
□	□	I/You feel it ominous seeking cancer screening.
□	□	I/You fear that cancer screening may damage my health and cost me too much.
□	□	I/You don’t want to upset/scare my family by telling them that I need cancer screening
□	□	I/You do not know where to get cancer screening.
□	□	I/You don’t know when to seek cancer screening.
□	□	I/You fear that cancer screening may take too long time and I have a tight time table.
□	□	I/You don’t know how to prepare for cancer screening.
□	□	It makes me and my family members worry too much if I were diagnosed with cancer.
□	□	I/You may be stigmatized if I were diagnosed with cancer.
□	□	Other (please enter)
Step 4: Counseling resource use and skills improvement (C4)		
S4a	Now, let’s discuss how to overcome these problems or barriers?	
□	□	[Many researches have proved that cancer screening greatly reduces cancer risks and harms.]
□	□	[Many of your peers, e.g., …, have been receiving regular screening and keep free from cancer for years.]
□	□	[Cancer screening does not harm to your health except minimum pain and uncomfortable experiences.]
□	□	[It costs a few hundred yuan depending only type and content of examinations/tests to be performed.]
□	□	[Most cancer screening expenditures can be claimed back from national insurance programs.]
□	□	[Talking about cancer screening with your family member(s) does much more benefits than harms.]
□	□	[It gains you various supports to implement cancer screening as well as other protective behaviors.]
□	□	[It also conveys useful information about cancer screening and prevention to your family member(s).]
□	□	[You can get cancer screening from any cancer specialty or general hospital of county level or over.]
□	□	[Here is a list of qualified hospitals that provides cancer screening and their contact details.]
□	□	[Here is a referral letter that tells why you need screening and what type of screening suits you most.]
□	□	[You need to get your first cancer screening as soon as possible.]
□	□	[Then you need to seek cancer screening every few years depending on results of the previous screening.]
□	□	[Cancer screening takes no longer than a half day and there are always ways to arrange such a time.]
□	□	[Medical checkup is always a justified reason asking for favor from relatives, friends, managers etc.]
□	□	[If in need, I would like to write you a letter as a proof for asking for such helps.]
□	□	[You needn’t any preparation except that you do not eat and drink 6 h before cancer screening.]
□	□	[You’d better ask accompany from a close relative or friend, which gives various supports and helps.]
□	□	[Negative screening result frees you and your relatives from worries rather than aggravates worries.]
□	□	[Even for those screened with positive results, they perceive the screening as a right rather than regretful decision, since it entails earlier treatment and better prognosis.]
□	□	[Doctors have obligations not to tell your diagnose to anyone else without your permission.]
□	□	[You may choose to disclose your diagnose to those you trust most or only yourself.]
□	□	Other (please enter)

Note: (1) Items without “[]” apply to all patients; while items within “[]” apply only to the specific patient under concern depending on his/her assessed need; (2) The left column check boxes (“□”) are used for checking ideas voiced by the patient independently; while the right column check boxes are used for checking viewpoints the patient agreed upon after he/she has get hints/advices from his/her counselor doctor

#### Rapid and detailed risk assessment

In order to identify high-risk farmers and thus deliver focused intervention, the study utilizes a two-stage assessment strategy, i.e., RRA followed by detailed risk assessment DRA. RRA takes about 10 min and covers all visiting patients aged 35+ who have not received RRA in the past 2 years. It solicits information about risks of developing cancer(s) for individual patients using a web-based 21-item structured questionnaire [22] and automatically produces, via the web-based system, a risk score for the patient. If the score were greater than the 70th percentile of all the RRA scores, a further 20–35 min DRA follows which expands the scope and detail of the information collected via the previous RRA using again a web-based structured instrument [22]. This DRA also automatically generates a risk score for each patient and if the DRA scored greater than the 80th percentile of all the DRA scores, the patient is eligible for receiving further intervention and/or evaluation.

Calculation of both the risk scores utilizes the formulae: a); b). Where *k* ranges from 1 to 9 standing for the nine most common cancers in rural China respectively; *P*_*k*_, age and gender-specific incidence rate of cancer *k* in rural China; *R*_*k*_, risk score of cancer *k* of the individual farmer under concern; *n*, the number of risk factors included in rapid (*n* = 164) and detailed (*n* = 157) risk assessment; *X*_*i*_, the Likert scale of the risk factor *X*_*i*_ generated via the rapid/detailed assessment; *W*_*ki*_, pooled odds ratio of cancer *k* for risk factor *i* derived through systematic review and meta-analysis of published researches on the same odds ratios among farmers in China; and *R*, total risk score of the farmer for developing any of the leading cancers.

#### Initial CS counseling

**Fig. 3:**
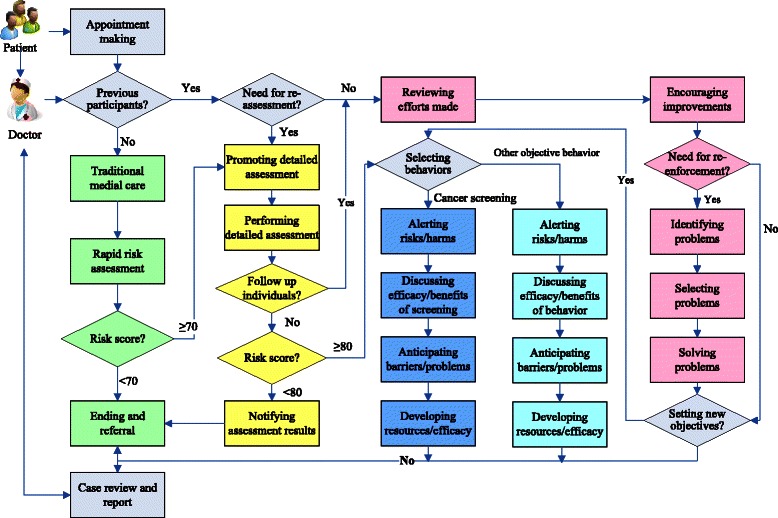
Flow-diagram of cancer screening promotion

Initial CS counseling applies to high risk farmers defined by the above mentioned rapid and detailed risk assessment (RRA ≥70th percentile of all RRA scores and DRA ≥ 80th percentile of all DRA scores respectively). The counseling takes about half an hour and follows SOPs developed under the guidance of the theoretical framework and profile mentioned earlier. The SOPs strive to promote regular CS use (O in Fig. 1) through 4 consecutive steps (blue rectangles in Fig. 3) each aims at improving one of the cognitive-affective components (C1 through to C4) in Fig. 1 respectively (Table 2). Step 1 makes the counselee farmer fully aware of his/her chances of getting cancer and harms the disease does to him/her. Step 2 raises his/her beliefs in the effectiveness and benefits of CS. Step 3 discusses probable barriers and problems he/she may encounter in seeking CS. Step 4 helps him/her identify or develop potential resources and self-efficacy for overcoming the barriers and problems.

#### CS reinforcement counseling

CS reinforcement counseling applies to farmers who have already received the abovementioned initial counseling and focuses on reinforcing behavior improvement and solving problems encountered in implementing the behavior changes. The counseling again takes about 30 munities and follows SOPs consisting of 3–7 consecutive steps (pink rectangles in Fig. 3). Step 1 examines what have the counselee done regarding CS since the last counseling session. Step 2 appreciates achievement made and encourages continuous efforts. Step 3 assesses whether the counselee needs further counseling on seeking regular CS and leads the counseling to either step 4 or step 7. Step 4 defines the problems encountered by the farmer in seeking CS. Step 5 helps the counselee select the most important yet resolvable problems to address for the next period. Step 6 provides necessary assistance for the farmer to solve the problems selected. Step 7 assesses whether the counselee needs to address additional objective behaviors and proceeds with relevant further SOPs.

CS reinforcement counseling is further divided into pre- and post-screening counseling. Pre-screening counseling happens once a month until the counselee has implemented the planned screening or stops after 5 consecutive counseling yet failed to reach its objective. Post-screening counseling takes place within two weeks after the counselee has completed a scheduled CS and aims at using the screening results to leverage further behavior changes and promote follow up screening.

#### Intervention workflow

Figure 3 depicts the main intervention procedures, the logic flows among these procedures and how they are integrated with traditional medical service at village clinics. For a given patient presenting to a village clinic, a self-developed smart web-aid for preventing cancer (SWAP-CA) automatically classifies (after inputting a unique identification number) the patient as participant or nonparticipant of the cancer prevention project or eCROPS-CA and then proposes SOPs for each kind of patient accordingly. If the patient is a nonparticipant, the system provides SOPs for performing the integrated rapid assessment introduced above, which in turn enables the system to automatically assign the patient as either high- or low-risk nonparticipant patient. For a high-risk nonparticipant patient, SWAP-CA leads to SOPs for promoting DRA, which further classifies the patient as high risk (DRA score ≥ the 80th percentile RRA score) or low risk (DRA score <80th percentile DRA score) patient. For a low risk patient, SWAP-CA tells the doctor to end the service for the patient. For a high risk patient, the system helps the doctor and patient to select one specific behavior from the pre-set objective behaviors of eCROPS-CA as mentioned earlier. If the selected objective behavior is CS, SWAP-CA proposes SOPs of the initial CS counseling described earlier. While for a patient who has received CS counseling for the last time, the system leads to SOPs of the CS reinforcement counseling.

Being an integral part of eCROPS-CA, how counseling for CS uptake is delivered in combination with that for other objective behaviors worth particular mentioning. Every high risk individuals identified via the aforementioned RRA and DRA in the intervention group is eligible for receiving SOPs for counseling part or all of the seven objective behaviors if applicable. Like CS counseling, counseling for each of the objective behaviors follows pre-set SOPs and comprises one initial and several reinforcement sessions depending on performance of the individual under concern. Each initial counseling session focuses on only one objective behavior. Specific objective behavior to be addressed for any given initial counseling session is determined by asking the counselee to select the first feasible behavior from a rank-order list of all non-addressed objective behaviors for that specific individual. The order list here is produced, by the SWAP-CA, in accordance with the relative contributions of the objective behaviors to the DRA score. This means that each initial counseling session addresses the then most feasible and important objective behavior for the specific individual and that the sequence of behaviors to be addressed varies from individuals to individuals.

### Delayed intervention

The delayed intervention arm maintains existing curative and preventive services without adding any prevention component included in eCROPS-CA except for RRA, DRA and planned project evaluation in the first 5 years.

### Study and data integrity

The study design follows CONSORT (Consolidated Standards of Reporting Trials) statement [33].

### Measures

The primary measures for assessing intervention efficacy are overall and specific CS uptake rates. Here overall CS uptake rate denotes percentage of farmers who have actually received any type of cancer screening to farmers who are eligible for the service during the past 12 months; while specific CS uptake rate (say breast cancer screening rate), percentages of farmers who have actually received screening for a specific type of cancer to farmers who are eligible for that specific screening during the past 12 months. The secondary measures concern perceptions of: a) susceptibility and seriousness of cancer; b) effectiveness and benefits of CS; c) barriers to and dis-benefits of seeking CS; d) ability, resources and self-efficacy utilizing CS (for detailed content and calculation of these measures, please refer to Additional file 2). In addition, the trial also collects related social demographic variables including age, gender, ethnicity, migration patterns, marital status, and education.

### Evaluation time points

Evaluation of this sub-trial coincides with evaluation of the umbrella intervention package and happens at baseline and semiannually after baseline. Each round of field data collection lasts for one week scheduled at the week before doctor training and the last week of the 6th, 12th, 18th, 24th, 30th, 36th, 42th, 48th, 54th, and 60th month after the baseline respectively. Both intervention and delayed intervention arms receive identical evaluation using same questionnaire, same field data collectors and same assessment time points.

### Data analyses

**Fig. 4:**
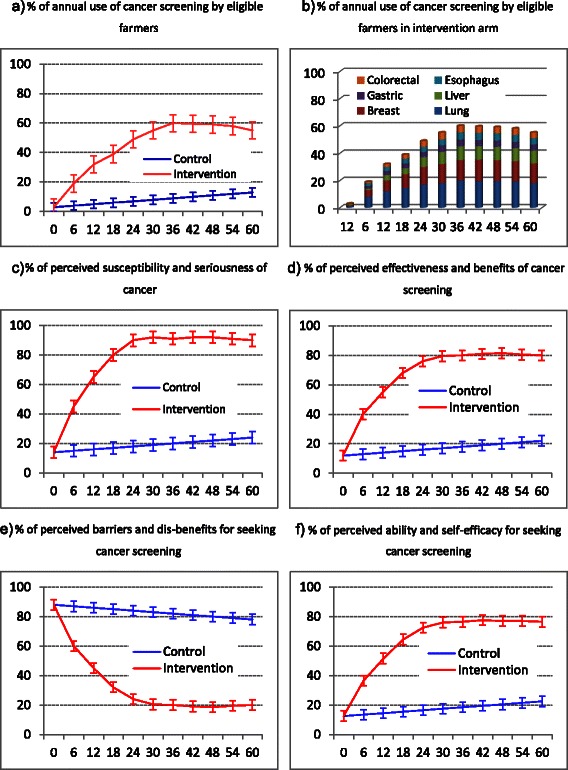
Anticipated outcome measures between intervention and control arms

Data analysis proceeds in four steps. Initial analysis centers on descriptive summaries intended to examine characteristics of the primary and secondary measures mentioned above and of subjects in intervention and delayed-intervention arms (Fig. 4). The next step estimates, using two-sided test of the null hypothesis, of the power of differences between the two arms and between different evaluation time points in terms of the two kinds of measures. The third step explores multivariate models, such as regression and path analysis between the primary measure (overall or specific CS uptake rate by different evaluation time points) and the secondary measures and socio-demographics of study subjects. The last step examines effects of counseling for other objective behaviors implemented by the umbrella project on CS uptake using again multivariate models between CS uptake at different time points and: a) perceptions of CS as well as other objective behaviors; b) changes (say from the previous to the current time point) in perceptions of CS as well as other objective behaviors.

### Ethics

This project involves recruitment, intervention and assessment of farmers and village doctors. So it adheres to rigorous human subject protection principles and procedures. The study protocol had been reviewed and approved by the Biomedical Ethics Committee of Anhui Medical University. Participation of farmers and village doctors are voluntary and written informed consent is sought from all participants.

## Discussion

The current trial distinguishes itself from previous ones because it not only adopts a personalized strategy but also proposes packaged solutions to tackling potential barriers and incorporating key success factors in a synergetic way toward low cost, effective and sustainable CS promotion. In addition to sharing most of the common features of its umbrella project as described separately [22], the first point worth noting with this CS promotion package refers to the theory-guided SOPs. Derived through evidence- and theory-based consensus, the SOPs should help both in ensuring delivery of key contents or steps of CS counseling and hence efficacy of the service and in simplifying the intervention procedures and hence reduction in delivery and training costs. With the combined guidance of the trans-theory framework (Fig. 1) and the determinant profile (Fig. 2), the SOPs developed incorporates key components of health belief model, motivational interviewing as well as our own research findings from local individuals [31]. Both health belief model and motivational interviewing have been applied successfully for leveraging behavior changes in various population groups [28, 29]. In development of the SOPs, these two theories served as references for generating key success factors to desired CS uptake; while the determinant profile based on qualitative interviews provided clues to what are most import in ensuring these key success factors as far as the specific local farmers were concerned. Counseling sessions reflecting both the profile and theories should be theoretically sound and socio-culturally sensitive.

Another point worth mentioning concerns theory-based focuses of counseling. These include motivation, cognitive dissonance, self-efficacy, as well as empathic processes. First, the counseling aims at raising motivation or commitment for the counselee to seek CS. It views motivation as a state of readiness for change rather than a personality trait that is relatively stable. Lack of motivation, therefore, is not a set individual characteristic but rather malleable [34]. Second, the CS counseling tries to produce a dissonant state first and then controls the direction chosen for the dissonance resolution. Cognitive dissonance defines the feeling of psychological discomfort produced by combined presence of two thoughts that do not follow from one another and being psychologically uncomfortable, the existence of dissonance motivates the individual to reduce it. The greater the discomfort is, the greater the desire to reduce the dissonance [35]. Third, the CS counseling strives to build self-efficacy, a person’s belief that he/she can carry out regular CS. People with high efficacy expect to succeed, realize favorable outcomes, holds beliefs that they can overcome obstacles by persevering and by improving self-management skills, does not give up in the face of difficulties and vice versa [36]. Forth, the CS counseling emphasizes accurate empathy via skillful reflective listening that clarifies and amplifies the counselee’s experience and meaning, without imposing the counselor’s own material. It communicates respect and caring, and builds a working alliance between counselor and counselee [37].

A third point of significance relates to the novel stage-wise cancer risk assessment instruments and models. Cancer happens at about 300 per 100,000 a year on average [38]. Such an incidence rate has important implications both for prevention planners and ordinary residents. For planners, it suggests that individual-based prevention against cancer targeting at non-selective subjects may not be cost-effective since the number needed to treat (NNT) is too big (>100,000/300) [39]; while for ordinary residents, the incidence rate makes it too easy to perceive low susceptibility since only less than 300 out of 100,000 could get cancer for a whole year [40]. According to our preliminary qualitative and quantitative surveys, by setting a proper cutoff score, the rapid and detailed risk assessment tools we had developed may help greatly in narrowing down the scale of intervention and thus in focusing scarce resources on the most needed. As specified earlier in the intervention schedule, although all visiting patients age 35+ need rapid risk assessment which takes only about 10 min, detailed risk assessment covers only 30 % of them and personalized CS promotion, only 6 %. More importantly, most of the patients scored with the top 6 % highest risk scores acknowledged that they were at elevated risk to develop cancer and needed to take action reducing their risks. In other words, the risk score can serve an effective means to promote CS and other objective behaviors.

The current trial also has limitations. Although the quasi-RCT design and the relatively large number of participants allow us to detect potential differencesbetween the intervention and the delayed intervention arms in terms of CS uptake rates and perceptions about CS, as a comprehensive intervention, it is hard to distinguish the effects of specific components within the package. The umbrella project, eCROPS-CA, strives to promote a series of objective behaviors and CS is one among them. In other words, a same farmer may receive multiple counseling sessions for different objective behaviors. Interactions between these interventions may pose problems telling effects of CS from that of the others, though the large trial scale and multiple time-point data collection allow for sub-group comparisons between combinations of interventions, e.g., CS promotion alone in intervention vs. delayed intervention conditions, promotion of CS plus other objective behaviors between the two groups, CS promotion alone vs. promotion of CS plus other objective behaviors. The multivariate modeling mentioned in the data analysis may also help in attributing the effects to CS counseling and interventions for promoting other objective behaviors. Besides, the cancer risk scores may generate fears among the assessed and the participating village doctors need adequate training on how to hind and address it.

## Additional files

Additional file 1: **Project subject sampling and randomization.** (DOCX 80 kb)
Additional file 2: **Questions for soliciting evaluation data and calculation of outcome measures.** (DOCX 26 kb)

## Abbreviations

CS: Cancer screening
SOPs: Standardized operation procedures
KAP: Knowledge, attitudes and practices
RCT: Quasi-randomized controlled trial
CDC: Center for Diseases Prevention and Control
RRA: Rapid risk assessments
DRA: Detailed risk assessments
SWAP-CA: Self-developed smart web-aid for preventing cancer
CONSORT: Consolidated Standards of Reporting Trials
NNT: Number needed to treat
