# Supplementary material for: Assessment and model guided cancer screening promotion by village doctors in China: a randomized controlled trial protocol
Source: BMC Cancer. 2015 Oct 12;15:674. doi: 10.1186/s12885-015-1688-9 (PMC4603763; doi:10.1186/s12885-015-1688-9)
Supplement: Additional file 1: — Project subject sampling and randomization. (DOCX 80 kb) [file 12885_2015_1688_MOESM1_ESM.docx]

Additional file 1: Project subject sampling and randomization.
