# Supplementary material for: Assessment and model guided cancer screening promotion by village doctors in China: a randomized controlled trial protocol
Source: BMC Cancer. 2015 Oct 12;15:674. doi: 10.1186/s12885-015-1688-9 (PMC4603763; doi:10.1186/s12885-015-1688-9)
Supplement: Additional file 2: — Questions for soliciting evaluation data and calculation of outcome measures. (DOCX 26 kb) [file 12885_2015_1688_MOESM2_ESM.docx]

**Additional file 2: Questions for soliciting evaluation data and calculation of outcome measures**

**Part A: Evaluation questions and value assignment**

Q1 Have you sought cancer screening during past 12 months?

□ Yes [X_1_=1] □ No [X_1_=0] (Skip to Q2)

Q1.1What types of cancer screening you have received?

□ Lung cancer [X_2_=1 if ticked, or 0 otherwise]

□ Breast cancer [X_3_=1 if ticked, or 0 otherwise]

□ Liver cancer [X_4_=1 if ticked, or 0 otherwise]

□ Gastric cancer [X_5_=1 if ticked, or 0 otherwise]

□ Esophagus cancer [X_6_=1 if ticked, or 0 otherwise]

□ Colorectal cancer [X_7_=1 if ticked, or 0 otherwise]

□ None of the above [X_8_=1 if ticked, or 0 otherwise]

Q2. Please tell me what harms do cancers do to human being?

| □ | It damages the organ it originates first [X_9_=1 if ticked, or 0 otherwise] |
| --- | --- |
| □ | It then metastases and invades various organs like the lung, brain, liver, bone etc [X_10_=1 if ticked, or 0 otherwise] |
| □ | It can cause various physical sufferings like pain, dysfunction, wasting syndrome etc [X_11_=1 if ticked, or 0 otherwise] |
| □ | It can cause various psychological sufferings like fears, anxiety, depression etc [X_12_=1 if ticked, or 0 otherwise] |
| □ | There are no-radical cures for most cancers and the disease has a high mortality [X_13_=1 if ticked, or 0 otherwise] |
| □ | Most cancer therapies are costly and have side effects [X_14_=1 if ticked, or 0 otherwise] |
| □ | It affects one’s work, study and business pursues [X_15_=1 if ticked, or 0 otherwise] |
| □ | It incurs economic burdens and psychological sufferings on family members and the beloved [X_16_=1 if ticked, or 0 otherwise] |
| □ | It may damage family relations [X_17_=1 if ticked, or 0 otherwise] |
| □ | It damages one’s image among and expectations by others [X_18_=1 if ticked, or 0 otherwise] |

Q3. How, do you think, are the chances for a general farmer in China to get cancer?

| □ | It’s easy to name friends/acquaintances diagnosed with cancer [X_19_=1 if ticked, or 0 otherwise] |
| --- | --- |
| □ | Everyone is susceptible to cancer [X_20_=1 if ticked, or 0 otherwise] |
| □ | Each year, 300 out of 100 thousand farmers get cancer [X_21_=1 if ticked, or 0 otherwise] |
| □ | One’s life time chances for getting cancer estimates over 21% [X_22_=1 if ticked, or 0 otherwise] |

Q4. How do you think of your own chances to get cancer and why?

| □ | I have elevated chances for getting lung cancer [X_23_=1 if ticked, or 0 otherwise] |
| --- | --- |
| □ | I have elevated chances for getting breast cancer [X_24_=1 if ticked, or 0 otherwise] |
| □ | I have elevated chances for getting liver cancer [X_25_=1 if ticked, or 0 otherwise] |
| □ | I have elevated chances for getting gastric cancer [X_26_=1 if ticked, or 0 otherwise] |
| □ | I have elevated chances for getting esophagus cancer [X_27_=1 if ticked, or 0 otherwise] |
| □ | I have elevated chances for getting colorectal cancer [X_28_=1 if ticked, or 0 otherwise] |
| □ | My latest cancer risk score is among the highest [X_29_=1 if ticked, or 0 otherwise] |
| □ | I have family member(s) who had diagnosed with gastric cancer [X_30_=1 if ticked, or 0 otherwise] |
| □ | I have been suffering from cancer related conditions for years [X_31_=1 if ticked, or 0 otherwise] |
| □ | I have unhealthy eating habits for years [X_32_=1 if ticked, or 0 otherwise] |
| □ | I have been a heavy alcohol drinker for years [X_33_=1 if ticked, or 0 otherwise] |
| □ | I have been smoking for years [X_34_=1 if ticked, or 0 otherwise] |

Q5. What, do you think, you can get from cancer screening?

| □ | Cancer screening can detect and correct the pre-cancerous conditions and thus prevent cancers [X_35_=1 if ticked, or 0 otherwise] |
| --- | --- |
| □ | At early stages, cancer cells confine within limited boundary and can be radically cleared, e.g., by surgery [X_36_=1 if ticked, or 0 otherwise] |
| □ | At late stages, cancer cells metastases to other organs and becomes hard to be cleared from human body [X_37_=1 if ticked, or 0 otherwise] |
| □ | The earlier the detection of cancer, the better the outcomes of cancer treatment[X_38_=1 if ticked, or 0 otherwise] |
| □ | Regular screening not only detects early cancer but also communicates knowledge about cancer[X_39_=1 if ticked, or 0 otherwise] |
| □ | Cancer screening also helps in finding and correcting other health problems [X_40_=1 if ticked, or 0 otherwise] |
| □ | Even negative screening result frees you and your relatives from worries [X_41_=1 if ticked, or 0 otherwise] |

Q6. What problems or barriers you may encounter in seeking cancer screening?

| □ | I feel it ominous seeking cancer screening [X_42_=1 if ticked, or 0 otherwise] |
| --- | --- |
| □ | I fear that cancer screening may damage my health [X_43_=1 if ticked, or 0 otherwise] |
| □ | I fear that cancer screening may cost me too much [X_44_=1 if ticked, or 0 otherwise] |
| □ | I don’t want to upset/scare my family by telling them that I need cancer screening [X_45_=1 if ticked, or 0 otherwise] |
| □ | I do not know where to get cancer screening [X_46_=1 if ticked, or 0 otherwise] |
| □ | I don’t know when to seek cancer screening [X_47_=1 if ticked, or 0 otherwise] |
| □ | I fear that cancer screening may take too long time and I have a tight time table [X_48_=1 if ticked, or 0 otherwise] |
| □ | I don’t know how to prepare for cancer screening [X_49_=1 if ticked, or 0 otherwise] |
| □ | It makes me and my family members worry too much if I were diagnosed with cancer [X_50_=1 if ticked, or 0 otherwise] |
| □ | I may be stigmatized if I were diagnosed with cancer [X_51_=1 if ticked, or 0 otherwise] |

Q7. If you need a cancer screening within one month, how confident that you will eventually realize it?

| □ | 5- totally confident [X_52_=5 if ticked] |
| --- | --- |
| □ | 4 [X_52_=4 if ticked] |
| □ | 3 [X_52_=3 if ticked] |
| □ | 2 [X_52_=2 if ticked] |
| □ | 1-least confident [X_52_=1 if ticked] |

**Part B: Calculation of outcome measures**

1. Overall CS uptake rate =100%×$\sum X_{1}$/n_1_. Here “n_1_” stands for total number of participants in project evaluation.
2. Lung CS uptake rate =100%×$\sum X_{2}$/n_2_. Here “n_2_” stands for total number of participants in project evaluation eligible for lung cancer screening.
3. Breast CS uptake rate =100%×$\sum X_{3}$/n_3_. Here “n_3_” stands for total number of participants in project evaluation eligible for breast cancer screening.
4. Liver CS uptake rate =100%×$\sum X_{4}$/n_4_. Here “n_4_” stands for total number of participants in project evaluation eligible for liver cancer screening.
5. Gastric CS uptake rate =100%×$\sum X_{5}$/n_5_. Here “n_5_” stands for total number of participants in project evaluation eligible for gastric cancer screening.
6. Esophagus CS uptake rate =100%×$\sum X_{6}$/n_6_. Here “n_6_” stands for total number of participants in project evaluation eligible for breast cancer screening.
7. Colorectal CS uptake rate =100%×$\sum X_{7}$/n_7_. Here “n_7_” stands for total number of participants in project evaluation eligible for colorectal cancer screening.
8. Perceptions of susceptibility and seriousness=100%×$\sum\sum_{i=9}^{34} X_{i}$/$\sum_{i=9}^{34} n_{i}$. Here n_i_ stands for the number of the participants in the project evaluation who are eligible for providing information on the i^th^ variable.
9. Perceptions of effectiveness and benefits =100%×$\sum\sum_{i=35}^{41} X_{i}$/$\sum_{i=35}^{41} n_{i}$. Here n_i_ stands for the number of the participants in the project evaluation who are eligible for providing information on the i^th^ variable.
10. Perceptions of barriers and dis-benefits =100%×$\sum\sum_{i=42}^{51} X_{i}$/$\sum_{i=42}^{51} n_{i}$. Here n_i_ stands for the number of the participants in the project evaluation who are eligible for providing information on the i^th^ variable.
11. Perceptions of ability and self-efficacy =100%×$\sum X_{52}$/5n_52_.Here n_52_ stands for the number of the participants in the project evaluation who are eligible for providing information on the 52^th^ variable.
